# From stem cell to egg cell

**DOI:** 10.7554/eLife.91998

**Published:** 2023-09-29

**Authors:** Noor M Kotb, Prashanth Rangan

**Affiliations:** 1 University at Albany, State University of New York Albany United States; 2 Department of Cell, Developmental, and Regenerative Biology, Black Family Stem Cell Institute, Icahn School of Medicine at Mount Sinai New York United States

**Keywords:** stem cell, germ cell, development, transposable element, gene silencing, *D. melanogaster*

## Abstract

Experiments on female fruit flies reveal more about the molecular mechanisms involved as germline stem cells transition to become egg cells.

**Related research article** Pang LY, DeLuca S, Zhu H, Urban JM, Spradling AC. 2023. Chromatin and gene expression changes during female *Drosophila* germline stem cell development illuminate the biology of highly potent stem cells. *eLife* **12**:RP90509. doi: 10.7554/eLife.90509.

Most stem cells are able to differentiate into a wide variety of cell types in a given organ system for the purposes of regeneration and repair (Morrison and Spradling, 2008). In the blood, for example, hematopoietic stem cells can give rise to red blood cells, various types of white blood cell, and a range of other blood cell types (Seita and Weissman, 2010). On the other hand, in the reproductive system, “germline” stem cells give rise to just two cell types – egg cells and sperm cells (Lehmann, 2012). However, when a sperm cell fertilizes an egg cell, the resulting zygote has the potential to give rise to an entire organism containing a large variety of cell types.

As a germline stem cell transitions into an egg cell, a delicate balance must be struck: genes specific to the stem cell must be silenced, and egg-specific genes must be activated. Once activated, these genes have a pivotal role in directing egg formation and ensuring a smooth transition from a germline stem cell to an egg cell. However, the mechanisms through which reproductive stem cells regulate this potential remain largely unknown.

Initially, it was believed that there existed a simple "on-off switch" between the stem cell and egg-specific programs. However, recent research has challenged this notion by showing that most of the genes essential for egg formation and development are expressed continuously during the stem cell stage, albeit at lower levels (Blatt et al., 2021; Sarkar et al., 2023; DeLuca et al., 2020). This suggests that, genetically, the egg program is always active in the mature reproductive organs, with stem cells restricting their potential to become eggs by ensuring that these genes are not expressed (Blatt et al., 2020; Slaidina and Lehmann, 2014). Thus, for genes that promote egg development, regulation is more similar to a "volume control" than a binary "on-off" switch.

Now, in eLife, Allan Spradling and colleagues at the Carnegie Institution for Science – including Liang-Yu Pang as first author – report the results of experiments on female fruit flies that reveal more details about how a germline stem cell becomes an egg cell (Figure 1; Pang et al., 2023).

**Figure 1.**
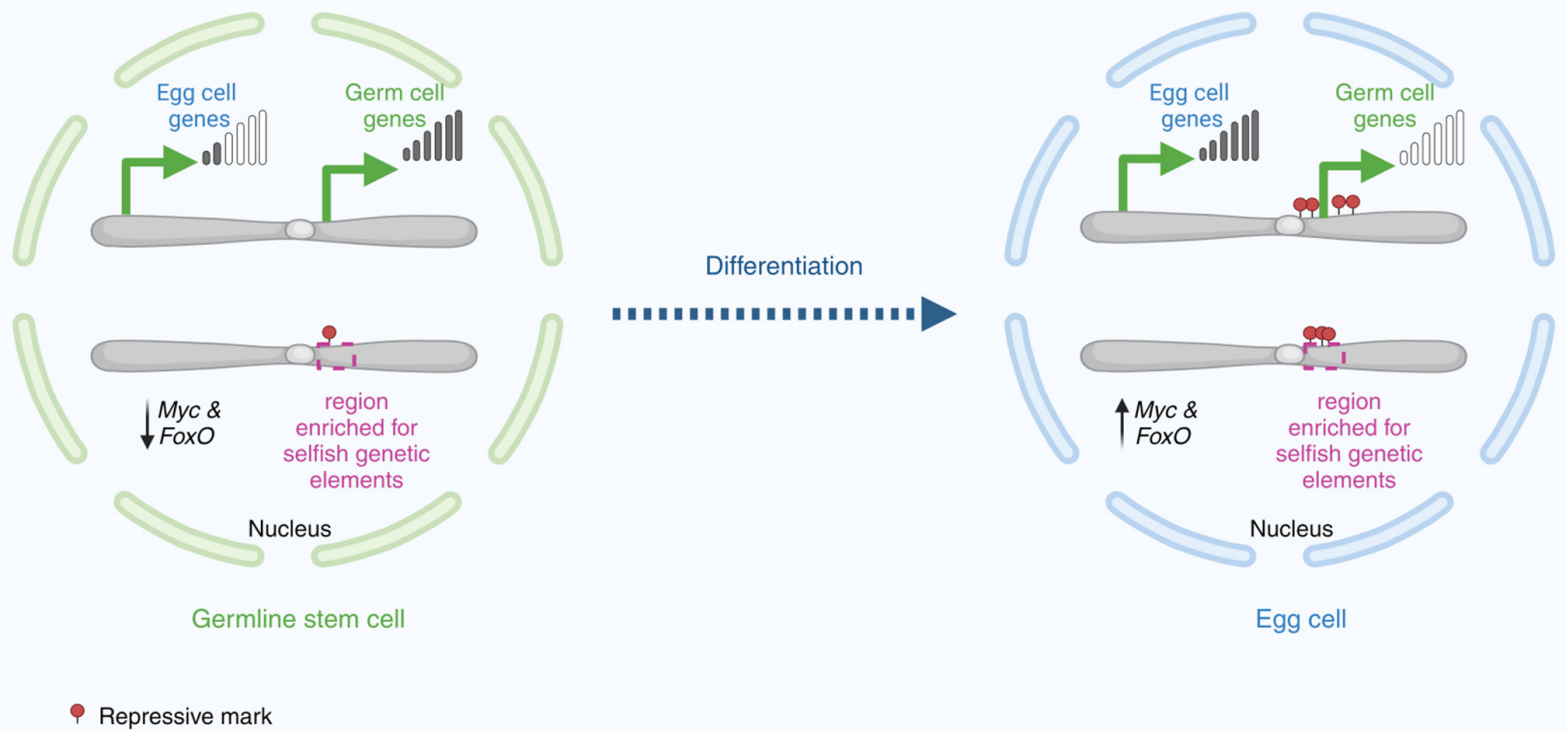
Changes in gene expression as a germline stem cell becomes an egg cell. In the nucleus of a germline stem cell (left), germ cell genes near the centre of the chromosome – adjacent to a region enriched with selfish genetic elements – are expressed at high levels. Conversely, egg cell genes and the transcription factors *Myc* and *FoxO* are transcribed at lower levels. As the germline stem cell differentiates to become an egg cell (right), Myc and FoxO levels increase to meet the metabolic demands required by the egg cell as it grows. In the nucleus, the egg cell genes are expressed at high levels, whereas the germ cell genes are silenced by histone modifications (red circles).

First, Pang et al. compared the gene expression of germline stem cells and egg cells. RNA sequencing revealed that most of the genes expressed in egg cells were also expressed at the earlier stage. However, the diversity of expressed genes decreased as the germline stem cells became egg cells.

Genes can be silenced when histones – proteins that interact with DNA and package it into a structure called chromatin – are modified by, for example, the addition of methyl groups to an amino acid residue in the histone. These modifications or ‘marks’ change how accessible genes are to the molecular machinery required to express them, and therefore regulate gene expression. Two marks, known as H3K9me3 and H3K27me3, are associated with gene silencing. A unit of chromatin contains eight histones, and H3K9me3 means that a histone called H3 has been modified by the addition of three methyl groups to the 9^th^ lysine (K) residue of this histone.

Using a combination of fluorescent imaging and a technique called ChIP-Seq, Pang et al. found that the number of both marks increase during the transition from germline stem cell to egg cell. Notably, the number of H3K9me3 marks increased in regions next to genes that support the stem cell program, and next to selfish genetic elements that can cause damage to the genome when activated. Loss of the enzyme responsible for depositing these marks upregulates genes supporting stem cell characteristics (Rangan et al., 2011; Sarkar et al., 2023; Sienski et al., 2015) and is required for proper egg production and fertility (DeLuca et al., 2020; Rangan et al., 2011). Similarly, loss of the enzymes required for H3K27me3 marks also upregulates stem cell genes. Taken together, these findings show that both H3K9me3 and H3K27me3 chromatin marks are necessary to completely silence the stem cell program and allow the transition to the egg cell to take place.

Egg growth requires significant metabolic changes and increased gene expression (Sieber and Spradling, 2017). Pang et al. observed upregulation of two transcription factors linked to increased metabolism and gene expression, *Myc* and *FoxO*, after the egg cell had formed. There were also large changes to mitochondrial metabolism that facilitated enhanced production of amino acids, nucleotides, carbohydrates, and lipids to support the substantial growth of the egg. Therefore, the transition from germline stem cell to egg cell is also accompanied by metabolic rewiring.

The work of Pang et al. sheds new light on our understanding of this transition from germline stem cell to egg cell, and raises further questions: we still do not know the identity of the genes responsible for orchestrating the metabolic shift, or fully understand the mechanisms that drive the upregulation of the egg program. Furthermore, it remains a mystery how stem cells exert control over selfish genetic elements when they are in an open chromatin state and able to be expressed. Research in this direction promises to unveil deeper insights into how stem cells modulate their ability to differentiate.

## References

[bib1] Blatt P, Martin ET, Breznak SM, Rangan P (2020). Post-transcriptional gene regulation regulates germline stem cell to oocyte transition during *Drosophila* oogenesis. Current Topics in Developmental Biology.

[bib2] Blatt P, Wong-Deyrup SW, McCarthy A, Breznak S, Hurton MD, Upadhyay M, Bennink B, Camacho J, Lee MT, Rangan P (2021). RNA degradation is required for the germ-cell to maternal transition in *Drosophila*. Current Biology.

[bib3] DeLuca SZ, Ghildiyal M, Pang LY, Spradling AC (2020). Differentiating *Drosophila* female germ cells initiate polycomb silencing by regulating PRC2-interacting proteins. eLife.

[bib4] Lehmann R (2012). Germline stem cells: origin and destiny. Cell Stem Cell.

[bib5] Morrison SJ, Spradling AC (2008). Stem cells and niches: mechanisms that promote stem cell maintenance throughout life. Cell.

[bib6] Pang LY, DeLuca S, Zhu H, Urban JM, Spradling AC (2023). Chromatin and gene expression changes during female *Drosophila* germline stem cell development illuminate the biology of highly potent stem cells. eLife.

[bib7] Rangan P, Malone CD, Navarro C, Newbold SP, Hayes PS, Sachidanandam R, Hannon GJ, Lehmann R (2011). piRNA production requires heterochromatin formation in *Drosophila*. Current Biology.

[bib8] Sarkar K, Kotb NM, Lemus A, Martin ET, McCarthy A, Camacho J, Iqbal A, Valm AM, Sammons MA, Rangan P (2023). A feedback loop between heterochromatin and the nucleopore complex controls germ-cell-to-oocyte transition during *Drosophila* oogenesis. Developmental Cell.

[bib9] Seita J, Weissman IL (2010). Hematopoietic stem cell: self-renewal versus differentiation. Wiley Interdisciplinary Reviews. Systems Biology and Medicine.

[bib10] Sieber MH, Spradling AC (2017). The role of metabolic states in development and disease. Current Opinion in Genetics & Development.

[bib11] Sienski G, Batki J, Senti KA, Dönertas D, Tirian L, Meixner K, Brennecke J (2015). Silencio/CG9754 connects the Piwi-piRNA complex to the cellular heterochromatin machinery. Genes & Development.

[bib12] Slaidina M, Lehmann R (2014). Translational control in germline stem cell development. The Journal of Cell Biology.

